# Causal associations between frailty and low back pain: a bidirectional two-sample mendelian randomization study

**DOI:** 10.1007/s40520-024-02843-2

**Published:** 2024-09-11

**Authors:** Zuying Liu, Jiaming Fan, Huilian Bu, Lijun Fu, Cong Li, Letian Ma, Cunlong Kong, Zhongyuan Lu, Xinxin Li, Jian Wang, Qingying Liu, Jingjing Yuan, Xiaochong Fan

**Affiliations:** 1 Department of Pain Medicine, the First Affiliated Hospital of Zhengzhou University, Zhengzhou, 450000 Henan Province China; 2 Henan Province International Joint Laboratory of Pain, Cognition and Emotion, Zhengzhou, 450000 Henan Province China; 3 Department of Anesthesiology, Pain and Perioperative Medicine, the First Affiliated Hospital of Zhengzhou University, Zhengzhou, 450000 Henan Province China; 4 Department of Human Anatomy, School of Basic Medical Sciences, Zhengzhou University, Zhengzhou, 450001 Henan Province China

**Keywords:** Frailty index, Low back pain, Mendelian randomization, Causality

## Abstract

**Background:**

Previous observational studies have revealed a potentially robust bidirectional relationship between frailty and low back pain (LBP). However, the precise causal relationship remains unclear.

**Methods:**

To examine the potential causal association between frailty and LBP, we conducted bidirectional two-sample Mendelian randomization analysis (MR) study. Genetic data on frailty index (FI) and LBP were acquired from publicly available genome-wide association studies (GWAS). Various MR methodologies were utilized, such as inverse variance weighting (IVW), weighted median, and MR-Egger, to evaluate causality. Additionally, sensitivity analyses were conducted to evaluate the robustness of the findings.

**Results:**

Genetically predicted higher FI (IVW, odds ratio [OR] = 1.66, 95% CI 1.17–2.36, *p* = 4.92E-03) was associated with a higher risk of LBP. As for the reverse direction, genetic liability to LBP showed consistent associations with a higher FI (IVW, OR = 1.13, 95% CI 1.07–1.19, *p* = 2.67E-05). The outcomes from various MR techniques and sensitivity analyses indicate the robustness of our findings.

**Conclusion:**

Our research findings provide additional evidence bolstering the bidirectional causal relationship between frailty and LBP.

**Supplementary Information:**

The online version contains supplementary material available at 10.1007/s40520-024-02843-2.

## Introduction

Low back pain (LBP) is characterized by pain and discomfort in the area beneath the ribcage, above the buttock crease, and between the mid-axillary lines, with or without associated leg pain [1]. According to a comprehensive systematic review of 165 studies conducted across 54 countries, the estimated point prevalence of LBP ranged from 11.9 to 13.9% [2]. LBP has emerged as one of the primary causes of disability and work absences worldwide [3]. It represents a significant public health concern and imposes a considerable economic burden on society [4, 5]. In the United States, the annual financial burden attributed to LBP surpasses $100 billion, encompassing expenses related to medical treatments, lost wages, and reduced productivity [6]. An increasing number of medical practice guidelines recommend multiple treatments to alleviate pain and mitigate its consequences in the management of LBP [7, 8]. Considering the substantial global prevalence and significant burden associated with LBP, there is an urgent imperative to elucidate potential causal risk factors for LBP.

Frailty denotes a multifaceted clinical syndrome characterized by diminished physiological capacity in multiple organs or systems, coupled with heightened vulnerability to stress [9]. With the aging of populations, frailty is progressively increasing on a global scale. It is associated with adverse health outcomes including multimorbidity, disability, and increased mortality rates [10]. The Frailty Index (FI) is acknowledged as a reliable and effective instrument for identifying individuals who are at risk of developing frailty [11]. It is a continuous metric that quantifies frailty based on the proportion of health deficits attributable to the ageing process as a proportion of all deficits. These deficits may manifest as symptoms, signs, diseases, disabilities or abnormalities, which can be identified through laboratory tests, radiological imaging or even social factors [12]. Previous studies have shown a significant bidirectional correlation between frailty and LBP. Over a 2-year period, a significant correlation was observed in an Asian population between frailty and the prevalence of LBP. The prevalence of LBP was significantly higher in both pre-frail and frail groups compared to healthy individuals [13]. Leopoldino et al. showed that in older adults with LBP, frailty led to more disability and lower physical status scores for quality of life [14]. Coyle et al. showed that older adults with LBP were more likely to be frail than those without LBP [15]. However, whether there is a causal relationship between frailty and LBP remains unclear, and there is an urgent need for large-sample randomized controlled trials (RCTs).

However, due to methodological challenges and ethical constraints, conducting RCTs might not be feasible. In such circumstances, Mendelian randomization (MR), an epidemiological research strategy, can be employed to assess the causal relationship between exposure and outcome [16]. This approach emulates the methodological design of RCT studies, providing high-level evidence when direct RCTs are difficult to conduct. In MR, single nucleotide polymorphisms (SNPs) are utilized as instrumental variables (IVs) to assess causal effects between exposure and outcome [17]. Since the genotypes are established during conception, MR is generally not susceptible to reverse causation or confounding factors [18, 19]. This advantage has led to wide utilization of MR methodology for inferring causality, particularly using publicly available data from genome-wide association studies (GWAS). Recent investigations into the connection between frailty and various diseases using MR [20, 21], no study has yet reported a causal relationship between frailty and LBP. Therefore, this study used a two-sample MR approach to assess the potential bidirectional causality between frailty and LBP by obtaining GWAS data on large-scale FI and LBP.

## Methods

### Study design

**Fig. 1:**
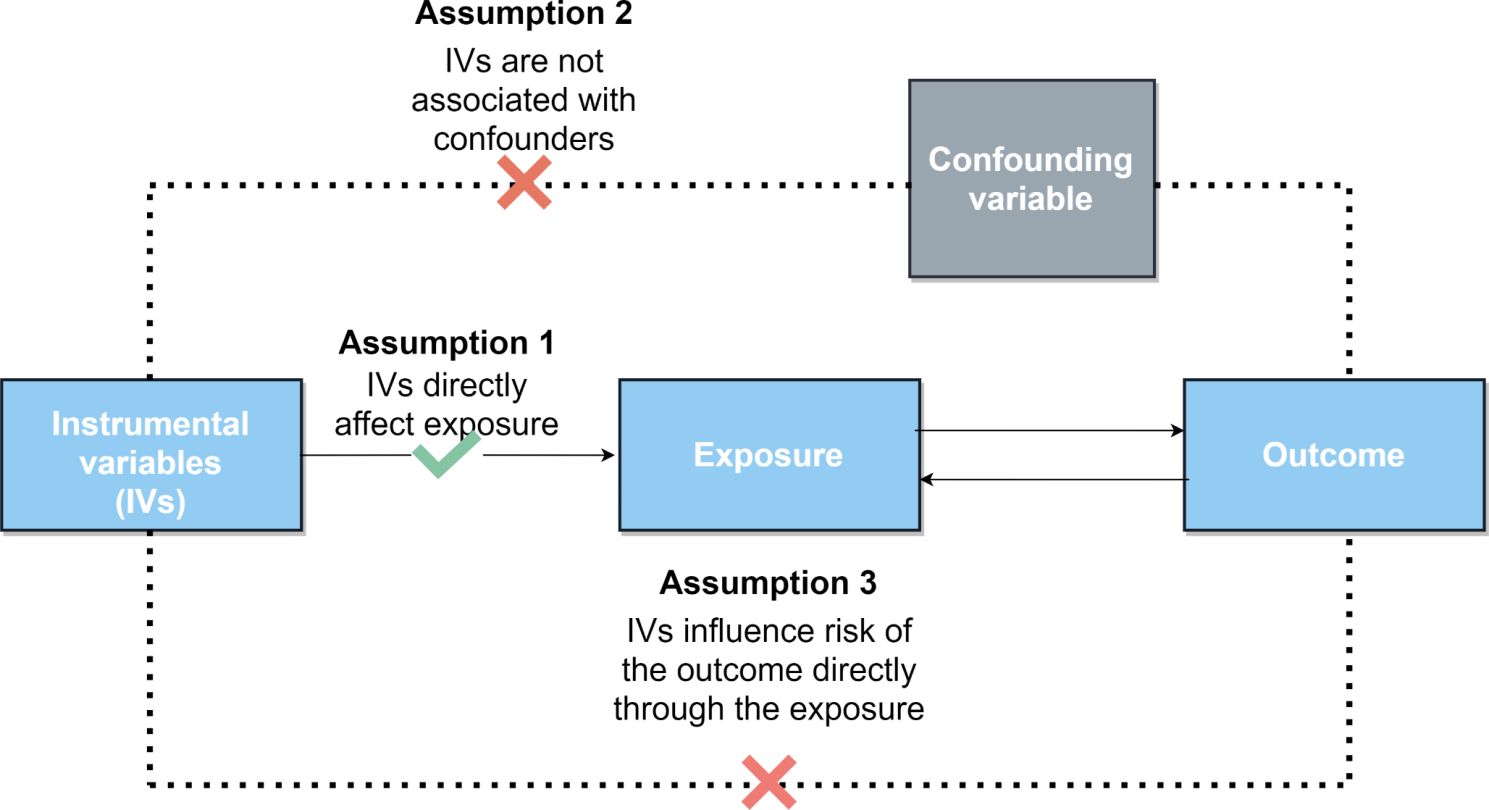
Overview of the bidirectional MR study design

Figure 1 presents an overview of the study design. This study utilized non-overlapping GWAS summary data within a standard two-sample MR framework to investigate the bidirectional causal relationship between frailty and LBP. All the data used in this study is publicly available, and the original study received ethical clearance and informed consent. MR is a data analysis technique utilized to assess etiological inferences in epidemiological studies. It employs genetic variants that demonstrate a strong correlation with exposure factors as IVs. The analysis relies on three fundamental assumptions: (1) The assumption of association: A robust association exists between SNPs and exposure factors. (2) The assumption of independence: Independence is present between SNPs and confounders. (3) The assumption of exclusivity: SNPs exclusively affect outcomes through exposure factors. These assumptions play a vital role in the accurate interpretation of causal relationships in MR studies.

### Data sources and genetic instrument selection

Detailed information is shown in Table 1. Summary statistics of frailty, assessed by the FI phenotype, were obtained through a comprehensive meta-analysis of GWAS conducted in the United Kingdom Biobank and Swedish TwinGene cohorts, which included 175,226 individuals of European ancestry [22]. The FI served as the proxy indicator of overall health; it is based on the accumulation of age-related deficits [23]. The FI was calculated on the basis of 49 and 44 self-reported items according to the cumulative error theory of the UK Biobank and TwinGene, respectively [22, 24]. The summary data for GWAS on LBP were obtained from the FinnGen dataset, which comprised 300,293 individuals of European ancestry. The identification of LBP was based on the International Classification of Diseases (ICD) codes obtained from nationwide registries in Finland (ICD10 - M54.5).

**Table 1:** Summary of GWAS included in this study

Year	Trait	Population	Cases	Controls	Samplesize	Websource
2021	Frailty	European	NA	NA	175,226	DOI: 10.1111/acel.13459
2023	Low back pain	European	29,329	270,964	300,293	www.finngen.fi/en

In conducting the MR analysis, we meticulously assessed the IVs used. To address the three main hypotheses of MR described earlier, we conducted a screening for SNPs that demonstrated a strong association with exposure using a stringent threshold (*p* < 5E-08) [25]. Additionally, we excluded weak IVs to prevent potential bias, including only IVs with an F statistic greater than 10. Furthermore, we performed clustering (r^2^ = 0.001, cluster distance = 10,000 kb) to address the potential bias caused by apparent linkage disequilibrium (LD) among the selected SNPs [26]. This step aimed to eliminate LD among the included IVs.

### Statistical analysis

Before conducting the MR analysis, we initially conducted the MR-Platform for Robust Estimation of Errors in Causality Testing (MR-PRESSO) test to identify any outliers. Following the identification and removal of these outliers, we proceeded with the MR analysis. The MR-PRESSO procedure was performed with a cycle number of 10,000 and *P* < 0.05 was used as a threshold to detect and remove outliers.

Given that inverse variance weighting (IVW) is known to offer accurate and stable results, we employed IVW as the principal analytical approach [27]. IVW is an extension of the Wald ratio estimator, founded on the principles of meta-analysis. Additionally, we employ the MR Egger and weighted median methods as supplementary approaches to MR. The variation in assumptions between these tests leads to a higher level of robustness when consistent effects are observed across multiple methods. The significance threshold was set at *p* < 0.05. A series of sensitivity analyses was subsequently conducted. The MR-Egger intercept test and Cochran’s Q statistic were employed to assess the presence of horizontal pleiotropy and heterogeneity, respectively [28, 29]. Additionally, a leave-one-out analysis was employed to assess the influence of individual SNPs on the overall estimates. The analyses in this study were conducted using R software (version 4.2.1). We utilized the “Two Sample MR” R package for our MR study [30].

## Results

### Instrumental variables for mendelian randomization

This study investigated the bidirectional causal relationship between frailty and the risk of LBP through two-sample MR. To assess the impact of frailty on the risk of LBP, we initially incorporated a set of 15 SNPs as IVs strongly linked to a FI. Furthermore, in the reverse MR analysis, we screened 11 SNPs as IVs specifically for LBP. All individual SNPs exhibited an F-statistic exceeding 10, signifying adequate instrumental strength. One SNP was lost when the outcome variable was merged. When investigating the effect of FI on the risk of developing LBP, we eliminated 2 SNPs by identifying outliers using MR-PRESSO, whereas no SNPs were eliminated at this step in reverse MR, and we finally included 12 and 10 SNPs, respectively, as IVs in the investigation. Tables S1 and S2 provide comprehensive details on the IVs, and Table S3 provides information on the outliers.

### The effect of frailty on the risk of low back pain

**Fig. 2:**
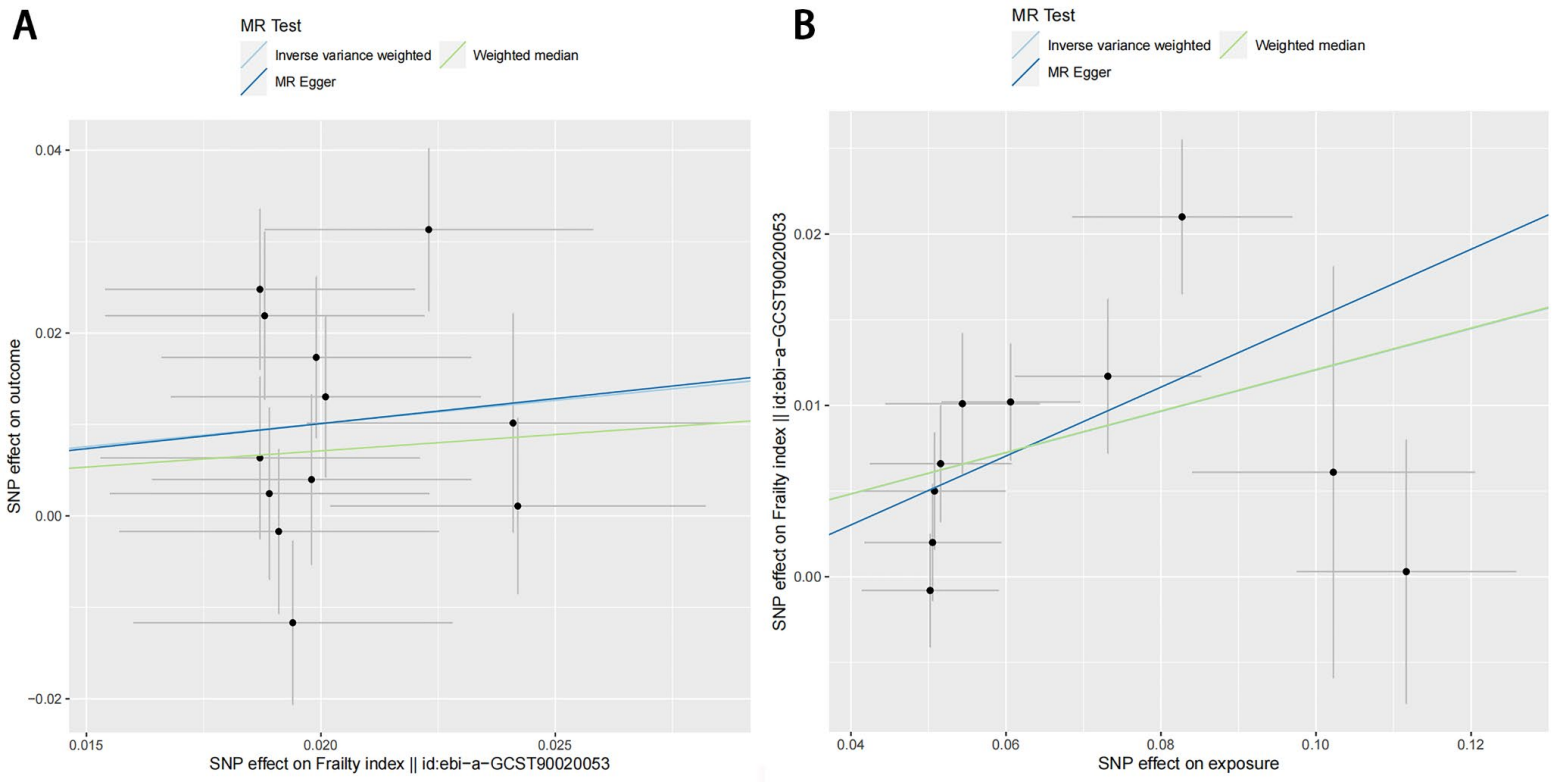
Scatter plots of single SNP effect and estimates from two-sample MR analyses for the causal effect of FI on LBP (**A**). Scatter plots of single SNP effect and estimates from two-sample MR analyses for the causal effect of LBP on FI (**B**)

The results of the MR analysis indicate a causal relationship between FI and LBP. According to the primary method of MR, the IVW results showed a significant association between genetically predicted higher FI.

and an increased risk of LBP (IVW, OR = 1.66, 95% CI 1.17–2.36, *p* = 4.92E-03; Table 2, Figure S1). Additionally, even though MR Egger and Weighted median did not yield consistent results compared to IVW, the beta values remained consistent across all methods (Fig. 2). Given the precision and robustness of IVW, we maintain a positive interpretation of the MR results. While Cochran’s Q statistic detected heterogeneity (Q = 20.60, *p* < 0.05, Table 3), the MR-Egger intercept suggested that horizontal pleiotropy did not influence the outcome in any analysis (intercept *p* value = -8.72E-04, *P* > 0.05, Table 3). Furthermore, the funnel plot (Figure S3) is symmetric, and the leave-one-out (Figure S2) results indicated that the MR results were not influenced by a single SNP.

**Table 2:** MR estimates from each method of assessing the bidirectional causal effects between frailty and low back pain

Exposure	Outcome	MR method	Number of SNPs	OR	SE	95% confidence interval	*P* value
Frailty	Low back pain	IVW	12	1.66	0.18	1.17–2.36	4.92E-03
		MR Egger	12	1.73	2.11	0.03-108.92	8.01E-01
		Weighted median	12	1.43	0.20	0.96–2.11	7.52E-02
Low back pain	Frailty	IVW	10	1.13	0.03	1.07–1.19	2.67E-05
		MR Egger	10	1.22	0.12	0.95–1.57	1.56E-01
		Weighted median	10	1.13	0.03	1.06–1.20	9.99E-05

**Table 3:** Sensitivity analysis of the MR analysis results of exposures and outcomes

Exposure	Outcome	Heterogeneity test	Pleiotropy test	MR-PRESSO	
		Cochran’s Q test (*P* value)	Egger intercept (*P* value)	Distorti-on test	Global test
		IVW	MR-egger	Outliers	*P* Value
Frailty	Low back pain	0.04	0.98	NA	0.06
Low back pain	Frailty	0.05	0.54	NA	0.08

### Results of reverse mendelian randomization analysis

In the reverse direction, there were significant associations between genetic liability to LBP and a higher FI (IVW, OR = 1.13, 95% CI 1.07–1.19, *p* = 2.67E-05; Table 2, Figure S1). The weighted median method yielded similar results. Although the results of MR Egger did not support the above hypothesis, we still conclude that LBP increases the risk of elevated FI. This conclusion is based on the lower precision of the MR-Egger method compared to other methods and the consistent direction of the beta value across all methods (Fig. 2). Cochran’s Q statistical test reveal significant heterogeneity in causality estimates among the IVs (Q = 16.96, *p* < 0.05, Table 3). However, the MR Egger intercept analysis found no evidence of directed pleiotropy (intercept *p* value = -0.005, *p* > 0.05, Table 3). Additionally, Additionally, The results of “leave one out” indicate that there is no single SNP that has a large role in driving the outcome (Figure S2). Additionally, the funnel plot provides further evidence that the study is unbiased (Figure S3).

## Discussion

A two-sample MR study was conducted utilizing the publicly available GWAS summary dataset to investigate the bidirectional causal relationship between the frailty and LBP. The MR analysis revealed a bidirectional causal relationship, where FI increased the risk of developing LBP, which in turn led to an increase in FI. This study is the first to assess the causal relationship between frailty and LBP using MR. These findings provide a theoretical basis for the development of management strategies targeting frailty and LBP in elderly patients.

Frailty and LBP are common issues in older adults that can severely impact their quality of life and overall health. Numerous epidemiological studies have examined the association between these two conditions. For instance, a 12-month longitudinal study involving 165 older adults suffering from LBP revealed that over two-thirds of the participants were classified as being either pre-frail or frail. Furthermore, the researchers observed that frailty was significantly linked with increased disability in older adults affected by LBP [31]. A longitudinal observational study of older adults in Brazil revealed a significant correlation between the degree of LBP and frailty [32]. Additionally, a prospective cohort study involving 602 individuals revealed that physical frailty was associated with increased pain intensity, lower scores in both physical and psychological aspects of quality of life, and higher disability scores among individuals with LBP [14]. It is important to acknowledge that prior research has been limited in its ability to determine causation of the relationship between frailty and LBP due to the susceptibility of observational studies to reverse causation and confounding variables. Our current study provides additional support of a bidirectional causal effect between FI and LBP, using a MR approach which is less susceptible to confounding bias than traditional observational designs. The discovery of this bidirectional relationship has important implications for public health and clinical practice. Frailty and LBP are reversible conditions with many modifiable factors. By understanding the relationship between frailty and LBP, risk factors can be proactively identified and appropriate interventions can be implemented. For example, older adults with LBP can receive regular assessments and treatment to reduce the likelihood of frailty. In addition to addressing LBP, it is important to focus on frailty management, including nutritional support, exercise rehabilitation, and psychological support. Ongoing early screening and treatment of frailty and LBP in older adults, along with the development of interventions to address common risk factors, can effectively reduce the adverse outcomes associated with frailty and improve the quality of life of older adults, which can play an important role in reducing the burden on society and families.

Several potential factors may explain the bidirectional causal relationship between frailty and LBP. First, frailty may lead to undesirable consequences such as falls, reduced endurance and altered morphology of the lumbar paravertebral muscles, and ultimately LBP [33, 34]. Additionally, frailty can lead to factors such as inadequate nutrition, sleep and mood disorders, increased healthcare expenses, and reduced social interaction, which may also contribute significantly to the development of LBP [35–37]. Conversely, mood and sleep disorders related to LBP may also increase the risk of frailty [38]. LBP has also been linked to cognitive impairment, which may further contribute to the development of frailty [39, 40]. Moreover, treatments that are effective for frailty and LBP can have synergistic benefits. For instance, physical activity not only enhances physical function in older and vulnerable populations, but also reduces pain and disability while improving quality of life in individuals with LBP [41, 42]. Therefore, the bidirectional relationship between frailty and LBP is not a random occurrence, and all of these findings provide support for this hypothesis. The current etiological model of the bidirectional causal relationship between frailty and LBP is too intricate to attribute to one or a few factors. Hence, additional research is imperative to investigate the specific mechanisms that underlie the bidirectional causality between frailty and LBP.

The present study aimed to elucidate the bidirectional causal relationship between frailty and LBP, and it highlights the following key aspects. Firstly, this study is the first of its kind to investigate the causal relationship between frailty and LBP utilizing a comprehensive GWAS pooled dataset. Secondly, our study employed multiple sensitivity analyses to test the hypotheses, thus enhancing the reliability of our results to a certain extent. Lastly, we employed MR analysis methods to minimize the impact of confounding factors, resulting in more accurate estimates in this study. However, there are limitations associated with our findings. Specifically, our analysis exclusively focused on a European population, which limits the generalizability of our results to other populations. Second, our utilization of summary datasets impeded our ability to stratify the data by gender, age, or other relevant factors. Third, it must be recognised that MR analyses are inherently less reliable than RCTs in providing evidence of causality. To address these limitations, future large-scale GWAS studies should be conducted across ethnic groups and differentiated by gender. There is also an urgent need for more high-quality RCT studies to confirm and strengthen these findings. However, investigating the causal relationship between frailty and LBP through RCTs is ethically challenging; therefore, extended prospective cohort studies may be a viable alternative. In addition, the effectiveness of interventions tailored to address common risk factors for frailty and LBP in improving patient care management warrants further investigation in future trials.

## Conclusion

This study provides support for a bidirectional causal relationship between frailty and LBP. Our findings suggest the importance of promoting frailty screening among patients with LBP. Furthermore, effective management of LBP is also crucial in mitigating the risk of frailty.

## Electronic supplementary material

Below is the link to the electronic supplementary material.

Supplementary Material 1

Supplementary Material 2

Supplementary Material 3

Supplementary Fig. 1. Forest plots of causal effects of FI on LBP (A). Forest plots of causal effects of LBP on FI (B). The bars indicate the confidence interval of MR estimates

Supplementary Fig. 2. Leave-one-out plots of two-sample MR analysis for genetically predicted FI and LBP (A) outcomes. Leave-one-out plots of two-sample MR analysis for genetically predicted LBP and FI (B) outcomes.The dots indicate MR estimates for using IVW method when the SNP was removed. The bars indicate the confidence interval of MR estimates

Supplementary Fig. 3. Funnel plots assess the presence of potential heterogeneity across genetic instruments for FI on LBP (A), which exhibited symmetry, indicating that the results were unbiased. Funnel plots assess the presence of potential heterogeneity across genetic instruments for LBP on FI (B), which exhibited symmetry, indicating that the results were unbiased. The causal effect of each genetic instrument was presented by dots, and combined causal effect by IVW and MR Egger were depicted by lines

## Data Availability

No datasets were generated or analysed during the current study.
