# Supplementary figures and images for: Causal associations between frailty and low back pain: a bidirectional two-sample mendelian randomization study
Source: Aging Clin Exp Res. 2024 Sep 11;36(1):191. doi: 10.1007/s40520-024-02843-2 (PMC11390933; doi:10.1007/s40520-024-02843-2)

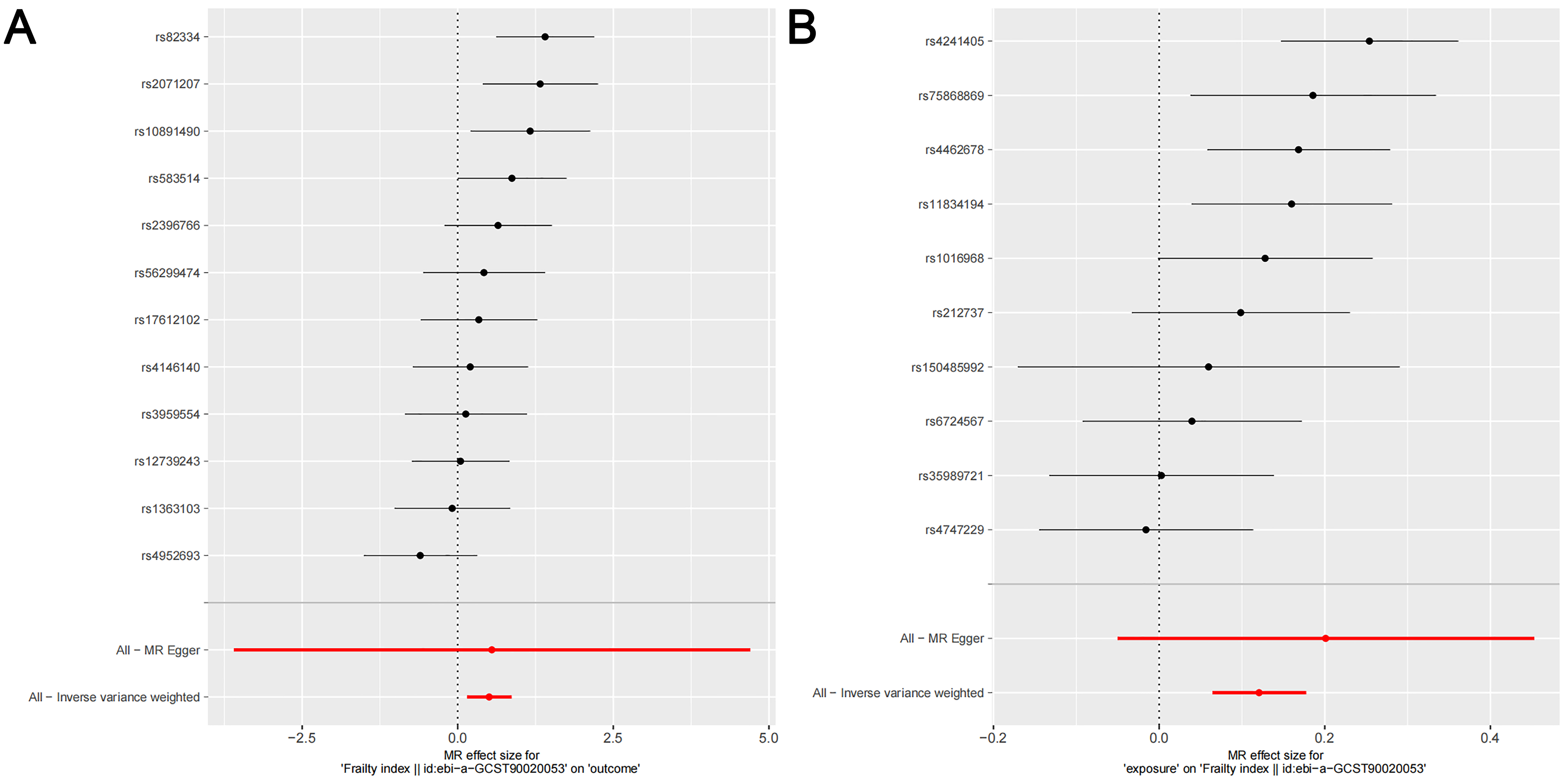

Supplement: Supplementary file 4 — Supplementary Fig. 1. Forest plots of causal effects of FI on LBP (A). Forest plots of causal effects of LBP on FI (B). The bars indicate the confidence interval of MR estimates [file 40520_2024_2843_MOESM4_ESM.tif]

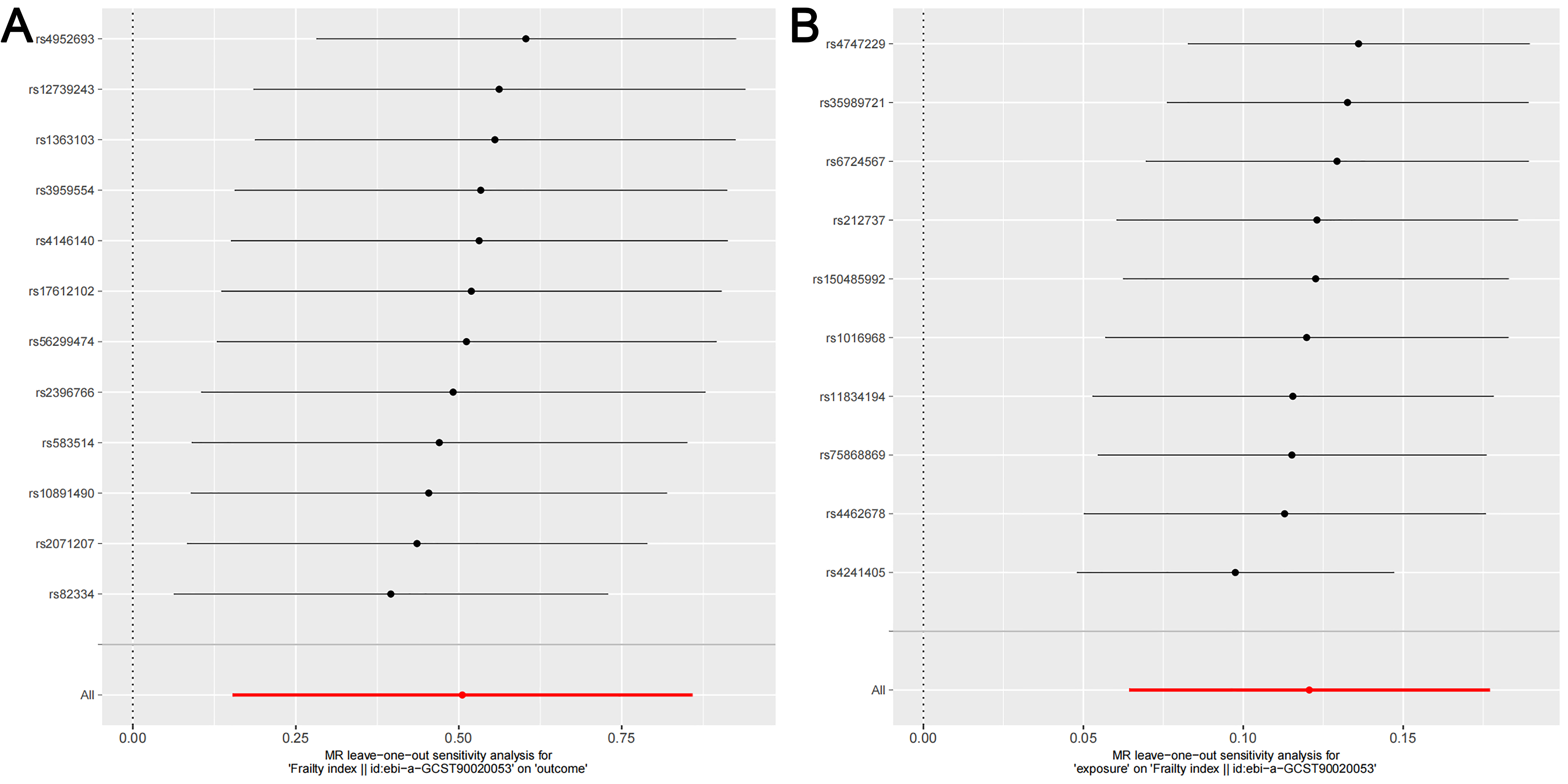

Supplement: Supplementary file 5 — Supplementary Fig. 2. Leave-one-out plots of two-sample MR analysis for genetically predicted FI and LBP (A) outcomes. Leave-one-out plots of two-sample MR analysis for genetically predicted LBP and FI (B) outcomes.The dots indicate MR estimates for using IVW method when the SNP was removed. The bars indicate the confidence interval of MR estimates [file 40520_2024_2843_MOESM5_ESM.tif]

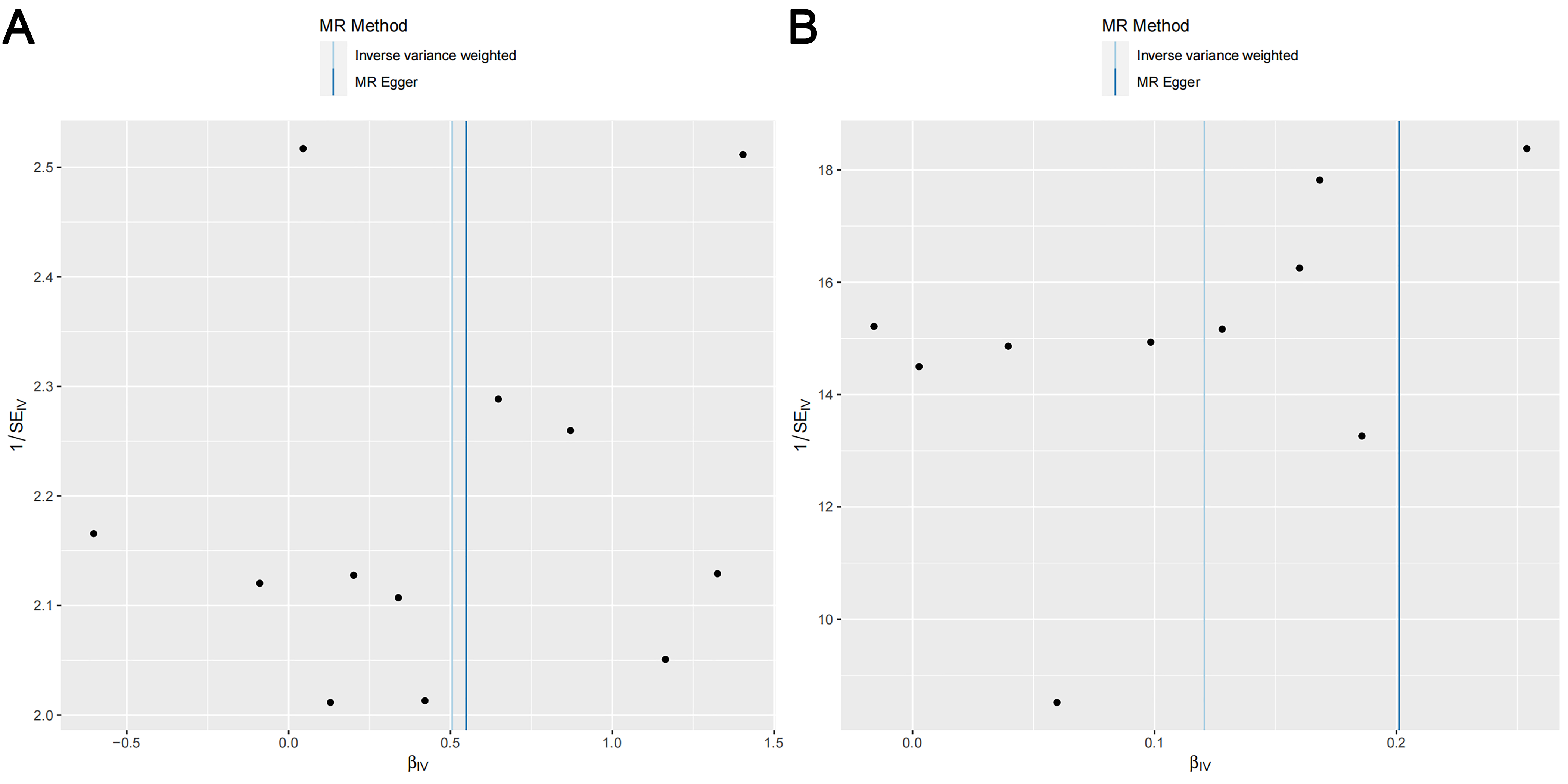

Supplement: Supplementary file 6 — Supplementary Fig. 3. Funnel plots assess the presence of potential heterogeneity across genetic instruments for FI on LBP (A), which exhibited symmetry, indicating that the results were unbiased. Funnel plots assess the presence of potential heterogeneity across genetic instruments for LBP on FI (B), which exhibited symmetry, indicating that the results were unbiased. The causal effect of each genetic instrument was presented by dots, and combined causal effect by IVW and MR Egger were depicted by lines [file 40520_2024_2843_MOESM6_ESM.tif]
